# Crystal structure of natural phaeosphaeride A

**DOI:** 10.1107/S205698901501395X

**Published:** 2015-07-31

**Authors:** Victoria V. Abzianidze, Ekaterina V. Poluektova, Ksenia P. Bolshakova, Taras L. Panikorovskii, Alexander S. Bogachenkov, Alexander O. Berestetskiy

**Affiliations:** a Chemical Modeling Laboratory, Research Institute of Hygiene, Occupational Pathology and Human Ecology, Medical Biological Agency, p/o Kuz’molovsky, Saint Petersburg, 188663, Russian Federation; b All-Russian Institute of Plant Protection, Russian Academy of Agricultural Sciences, Pushkin, Saint Petersburg, 196608, Russian Federation; c Department of Crystallography, Institute of Earth Sciences, Saint Petersburg State University, University Emb., 7/9, Saint Petersburg, 199034, Russian Federation; d Department of Organic Chemistry, Institute of Chemistry, Saint Petersburg State University, University Emb., 26, Saint Petersburg, 198504, Russian Federation

**Keywords:** crystal structure, natural phaeosphaeride A

## Abstract

The asymmetric unit of the title compound, C_15_H_23_NO_5_, contains two independent mol­ecules. Phaeosphaeride A contains two primary sections, an alkyl chain consisting of five C atoms and a cyclic system consisting of fused five- and six-membered rings with attached substituents. In the crystal, the mol­ecules form layered structures. Nearly planar sheets, parallel to the (001) plane, form bilayers of two-dimensional hydrogen-bonded networks with the hy­droxy groups located on the inter­ior of the bilayer sheets. The network is constructed primarily of four O—H⋯O hydrogen bonds, which form a zigzag pattern in the (001) plane. The butyl chains inter­digitate with the butyl chains on adjacent sheets. The crystal was twinned by a twofold rotation about the *c* axis, with refined major–minor occupancy fractions of 0.718 (6):0.282 (6).

## Related literature   

For details of the extraction of natural phaeosphaeride A and a discussion of its biological activities, see: Maloney *et al.* (2006 ▸). For details of trials of the synthesis of natural phaeosphaeride A, see: Kobayashi *et al.* (2011 ▸); Chatzimpaloglou *et al.* (2012 ▸, 2014 ▸); Kobayashi *et al.* (2015 ▸). Ring-puckering parameters are as defined by Cremer & Pople (1975 ▸). Hydrogen bonding is described in detail by Desiraju & Steiner (1999 ▸) and by Arunan *et al.* (2011 ▸). The twin law was identified using TwinRotMat in *PLATON* (Spek, 2009 ▸). Criteria for absolute configuration determination are described by Flack (1983 ▸) and Parsons *et al.* (2013 ▸).
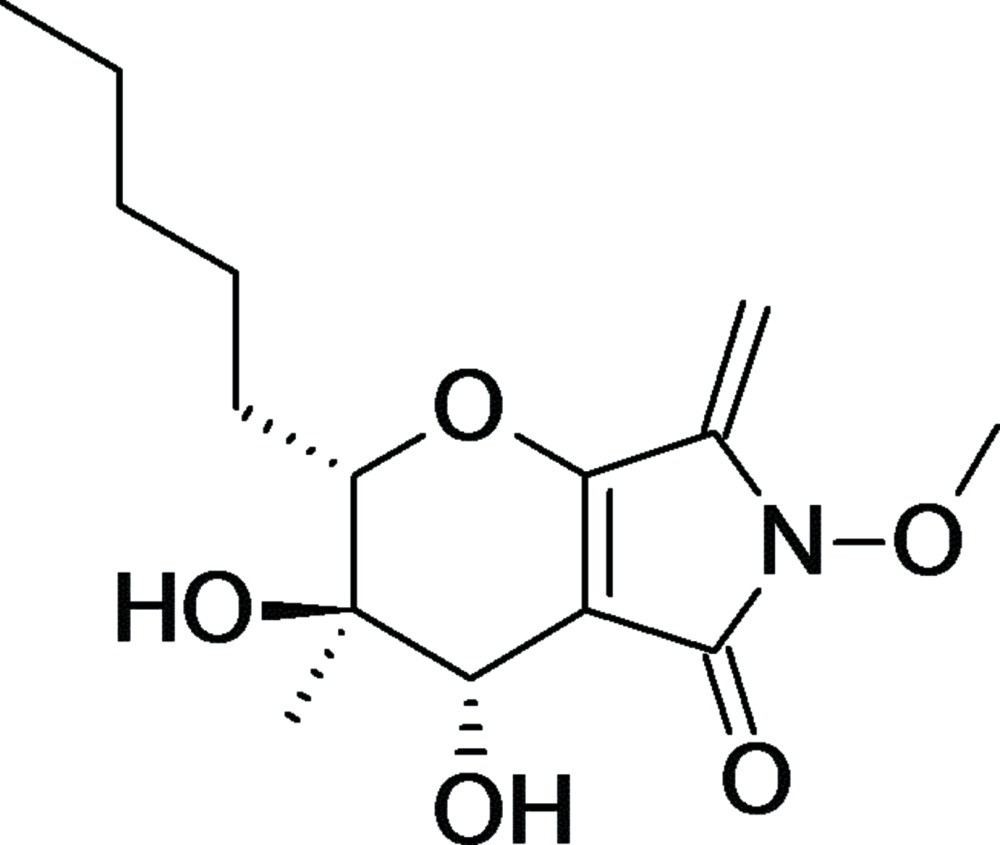


## Experimental   

### Crystal data  

C_15_H_23_NO_5_
*M* *_r_* = 297.34
Monoclinic, 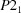

*a* = 10.14078 (18) Å
*b* = 9.10361 (14) Å
*c* = 17.5991 (3) Å
β = 100.1847 (16)°
*V* = 1599.11 (5) Å^3^
*Z* = 4
Cu *K*α radiation
μ = 0.77 mm^−1^
*T* = 100 K
0.35 × 0.35 × 0.05 mm

### Data collection  

Agilent SuperNova Dual Source diffractometer with an Atlas detector
Absorption correction: multi-scan (*CrysAlis PRO*; Agilent, 2012 ▸) *T*_min_ = 0.824, *T*_max_ = 1.000
6054 measured reflections
6054 independent reflections
5940 reflections with *I* > 2σ(*I*)

### Refinement  

*R*[*F* ^2^ > 2σ(*F* ^2^)] = 0.058
*wR*(*F* ^2^) = 0.150
*S* = 1.10
6054 reflections
389 parameters
1 restraint
H-atom parameters constrained
Δρ_max_ = 0.36 e Å^−3^
Δρ_min_ = −0.30 e Å^−3^
Absolute structure: Flack *x* determined using 2632 quotients [(*I* ^+^) − (*I* ^−^)]/[(*I* ^+^) + (*I* ^−^)] (Parsons *et al.*, 2013 ▸)
Absolute structure parameter: 0.05 (8)

### 

Data collection: *CrysAlis PRO* (Agilent, 2012 ▸); cell refinement: *CrysAlis PRO*; data reduction: *CrysAlis PRO*; program(s) used to solve structure: *SHELXS97* (Sheldrick, 2008 ▸); program(s) used to refine structure: *SHELXL2014* (Sheldrick, 2015 ▸); molecular graphics: *OLEX2* (Dolomanov *et al.*, 2009 ▸); software used to prepare material for publication: *OLEX2*.

## Supplementary Material

Crystal structure: contains datablock(s) I. DOI: 10.1107/S205698901501395X/pk2560sup1.cif

Structure factors: contains datablock(s) I. DOI: 10.1107/S205698901501395X/pk2560Isup2.hkl

**Fig. 1.**
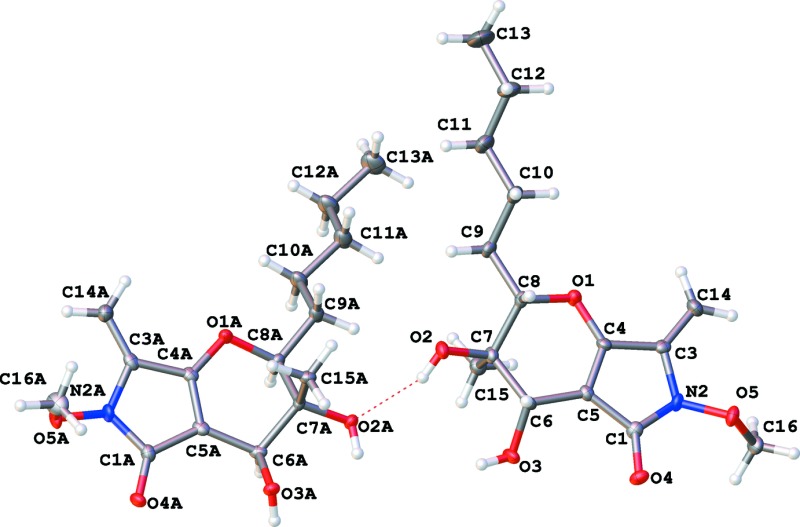
A view of molecules I (left) and II (right) of phaeosphaeride A. The atom numbering scheme is that of Maloney et al. (2006). Displacement ellipsoids are shown at the 50% probability level.

Supporting information file. DOI: 10.1107/S205698901501395X/pk2560Isup3.cml

et al. . DOI: 10.1107/S205698901501395X/pk2560fig1.tif
A view of mol­ecules I (left) and II (right) of phaeosphaeride A. The atom numbering scheme is that of Maloney *et al.* (2006). Displacement ellipsoids are shown at the 50% probability level.

**Fig. 2.**
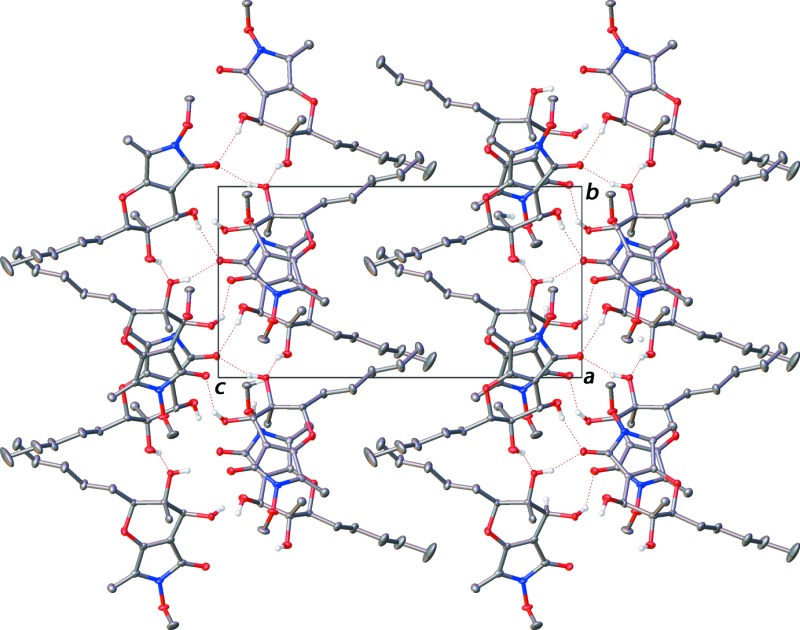
Projection of the layered crystal structure of phaeosphaeride A on the (100) plane. The dashed lines indicate the short contacts between molecules of phaeosphaeride A (only hydrogen atoms forming hydrogen bonds are shown).

. DOI: 10.1107/S205698901501395X/pk2560fig2.tif
Projection of the layered crystal structure of phaeosphaeride A on the (100) plane. The dashed lines indicate the short contacts between mol­ecules of phaeosphaeride A (only hydrogen atoms forming hydrogen bonds are shown).

CCDC reference: 1412515

Additional supporting information:  crystallographic information; 3D view; checkCIF report

## Figures and Tables

**Table 1:** Hydrogen-bond geometry (, )

*D*H*A*	*D*H	H*A*	*D* *A*	*D*H*A*
O2H2O2*A*	0.82	2.04	2.818(5)	158
O3H3O4^i^	0.82	2.03	2.836(5)	168
O2*A*H2*A*O4^i^	0.82	2.00	2.685(5)	141
O3*A*H3*A*O4*A* ^ii^	0.82	2.10	2.829(5)	149

Symmetry codes: (i) 
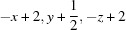
; (ii) 
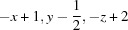
.

## supplementary crystallographic information

### S1. Comment

In 2006, Clardy and colleagues isolated phaeosphaeride A from an endophytic fungus FA39 (Maloney *et al.*, 2006). Phaeosphaeride A turned out to be an inhibitor of signal transduction and an activator of transcription 3 (STAT3)-dependent signaling. It was reported to selectively inhibit STAT3/DNA binding with an IC_50_ of 0.61 mM and to exhibit promising cell growth inhibition in STAT3-dependent U266 multiple myeloma cells with an IC_50_ of 6.7 µM.

While the relative stereochemistry of phaeosphaeride A was deduced on the basis of NOE experiments, its absolute configuration remained undetermined (Maloney *et al.*, 2006). Moreover, the attempts of total synthesis of phaeosphaeride A showed considerable differences in ^1^H and ^13^C NMR data between the synthetic and natural phaeosphaeride A (Kobayashi *et al.*, 2011; Chatzimpaloglou *et al.*, 2012, 2014). In 2015, Kobayashi and colleagues established the relative and absolute configurations of natural phaeosphaeride A by completing the first total synthesis of ent-phaeosphaeride A (Kobayashi *et al.*, 2015).

Our research group isolated phaeosphaeride A from a fungal strain belonging to the genus Phoma. Phaeosphaeride A was obtained as an optically active (-108.33 (c 0.06, CH_2_Cl_2_)) yellow glass. ^1^H and ^13^C NMR data as well as mass spectra of our phaeosphaeride A match with the data reported for Clardy's natural phaeosphaeride A (Maloney *et al.*, 2006). Optical rotation of Clardy's product (-93.6 (c 2.0, CH_2_Cl_2_)) and our phaeosphaeride A have the same sign. In this work we describe the crystal structure of natural phaeosphaeride A.

### S2. Experimental

NMR spectra were recorded on a Bruker AVANCE III 400 MHz spectrometer in DMSO-d6. The same solvent was used as an inter­nal standard. High-resolution mass spectra (HRMS) were recorded on a LTQ Orbitrap Velos spectrometer. Optical rotations were determined on an Optical Activity AA-55 polarimeter using a 20 cm cell with a Na 589 nm filter.

Phaeosphaeride A was isolated from solid culture of the fungus Phoma sp. N 19. The microorganism was obtained from leaves of Cirsium arvense (L.) Scop. and deposited in the culture collection of the All-Russian Institute of Plant Protection (Saint-Petersburg, Russian Federation). The metabolite was purified from the fungal extract with a combination of preparative column chromatography and TLC on silica gel to give phaeosphaeride A as a yellow precipitate. (-108.33 (c 0.06, CH_2_Cl_2_); ^1^H NMR (400 MHz, DMSO-d6) δ 5.44 (d, J = 5.8 Hz, 1H), 4.97 (s, 2H), 4.92 (s, 1H), 4.07 (d, J = 11.3 Hz, 1H), 3.86 (d, J = 5.8 Hz, 1H), 3.79 (s, 3H), 1.82 (m, 1H), 1.58-1.22 (m, 7H), 1.19 (s, 3H), 0.86 (t, J = 6.4 Hz, 3H); ^13^C NMR (100.6 MHz, DMSO-d6) δ 166.53, 155.30, 137.12, 104.80, 90.80, 86.25, 70.96, 64.36, 63.76, 30.90, 27.60, 26.11, 21.96, 20.40, 13.85; HRMS [M + H]+ calcd for C_15_H_24_NO_5_ 298.16490, found 298.16493. Recrystallization from heptane yielded yellow crystals (-116.66; -108.33 (c 0.06, CH_2_Cl_2_).

### S3. Refinement

Crystal data, data collection and structure refinement details are summarized in Table 1.

H atoms bonded to C atoms were included in calculated positions and refined using a riding model, with U*_iso_*(H) set to 1.2U*_eq_*(C) and C–H = 0.97 Å for CH_2_ groups, U*_iso_*(H) set to 1.5U*_eq_*(N) and C–H = 0.96 Å for CH_3_ groups and U*_iso_*(H) set to 1.2U*_eq_*(N) and C–H = 0.93 Å for CH groups. All H atoms bonded to O atoms were located in a difference Fourier map and were refined with distance restraints and constrained displacement parameters OH 0.82 Å and U*_iso_*(H) set to 1.2U*_eq_*(O). The large thermal ellipsoid on C13 is characteristic for the distal end of long alkyl chains.

The structure of phaeosphaeride A (Fig.1) was refined as rotational twin [by a two-fold rotation about (001)] with twin fractions of 0.718 (6) and 0.282 (6). The 'HKLF 5' format file for the final refinement was generated by the TwinRotMat facility in Platon (Spek, 2009). The ratio (F_c_^2^-F_o_^2^)/esd for the reflections with the highest error in final refinement model (as a rotational twin) has lower residuals than in the initial solution. We have used Bayesian Statistics for verifying absolute structure. Supplementary crystallographic data for this paper have been deposited at Cambridge Crystallographic Data Centre (CCDC 1412515) and can be obtained free of charge via www.ccdc.cam.ac.uk/data_request/cif.

### S4. Geometry

The asymmetric unit contains two independent molecules of phaeosphaeride A (I and II) (fig. 1). Each molecule of phaeosphaeride A (numbering of atoms of phaeosphaeride A is given according to Clardy (Maloney *et al.*, 2006)*)* contains two primary sections; an alkyl chain consisting of C(13)—C(12)—C(11)—C(10)—C(9) atoms and a cyclic system consisting of five and six membered rings with adjacent atoms.

For the five-membered ring C(1)—N(2)—C(3)—C(4)—C(5) of molecule I the Cremer-Pople (Cremer & Pople, 1975) parameters are Q=0.0714 Å, φ=197.99°, revealing a slightly distorted half-chair (^2^T_1_) conformation. Cremer-Pople parameters *of* Q=0.501 Å, θ=128.71°, φ=88.01° for the six-membered heterocycle of molecule I are consistent with a half-chair conformation (^5^H_4_). The geometric parameters of the six-membered rings for both molecules are similar, but the five-membered rings have different conformations. The five-membered ring in molecule II exhibits an envelope (^1^E) conformation.

Torsion angles O(1)—C(4)—C(5)—C(1) and C(3)—C(4)—C(5)—C(6) are -178.2 (4)° and -177.1 (4)° respectively, corresponding to co-planar conformation between the five and six-membered rings. The geometry of the heterocyclic ring system of the molecule (base of the half chair and the five-membered ring) is close to planar.

The exocyclic alkyl chain of I and the back of the half-chair lie approximately in the same plane. The deviation between the plane of alkyl atoms C(8)—C(9)—C(10)—C(11)—C(12)—C(13) and the back of the chair is 9.7 (4)°.

The geometric parameters are similar for both molecules I and II forming the weak hydrogen-bonded dimer through O2—H2···O2A. But angles characterizing the meth­oxy groups N(2)—O(5)—C(16) are slightly different (109.9 (3)° for I and 110.4 (4)° for II). The difference between angles O(4)—C(1)—C(5) is much greater with values of 129.4 (4)° and 123.2 (4)° for I and II respectively.

The molecules form layered structures. Nearly planar sheets, parallel with the (001) plane, form primary layers of two-dimensional hydrogen-bonded networks with the hydroxyl moieties located on the inter­ior of the sheets. The network (Fig. 2) is dependent primarily on four hydrogen bonds (Table 1) O2—H2···O2A, O3—H3···O4, O2A—H2A···O4, O3A—H3A···O4A (Desiraju *et al.*, 1999; Arunan *et al.*, 2011). In the (001) plane, two-dimensional hydrogen-bonded networks form a zig-zag pattern. The aliphatic butyl chains inter­digitate with the butyl chains on the adjacent sheets.

### Crystal data

**Table:**

C_15_H_23_NO_5_	*F*(000) = 640
*M**_r_* = 297.34	*D*_x_ = 1.235 Mg m^−^^3^
Monoclinic, *P*2_1_	Cu *K*α radiation, λ = 1.54184 Å
*a* = 10.14078 (18) Å	Cell parameters from 14376 reflections
*b* = 9.10361 (14) Å	θ = 4.4–75.9°
*c* = 17.5991 (3) Å	µ = 0.77 mm^−^^1^
β = 100.1847 (16)°	*T* = 100 K
*V* = 1599.11 (5) Å^3^	Tabular, colourless
*Z* = 4	0.35 × 0.35 × 0.05 mm

### Data collection

**Table:**

Agilent SuperNova Dual Source diffractometer with an Atlas detector	6054 measured reflections
Radiation source: SuperNova (Cu) X-ray Source	6054 independent reflections
Mirror monochromator	5940 reflections with *I* > 2σ(*I*)
Detector resolution: 10.3829 pixels mm^-1^	θ_max_ = 70.0°, θ_min_ = 4.4°
ω scans	*h* = −12→12
Absorption correction: multi-scan (*CrysAlis PRO*; Agilent, 2012)	*k* = −11→11
*T*_min_ = 0.824, *T*_max_ = 1.000	*l* = −4→21

### Refinement

**Table:**

Refinement on *F*^2^	Hydrogen site location: inferred from neighbouring sites
Least-squares matrix: full	H-atom parameters constrained
*R*[*F*^2^ > 2σ(*F*^2^)] = 0.058	*w* = 1/[σ^2^(*F*_o_^2^) + (0.0289*P*)^2^ + 2.9758*P*] where *P* = (*F*_o_^2^ + 2*F*_c_^2^)/3
*wR*(*F*^2^) = 0.150	(Δ/σ)_max_ < 0.001
*S* = 1.10	Δρ_max_ = 0.36 e Å^−^^3^
6054 reflections	Δρ_min_ = −0.30 e Å^−^^3^
389 parameters	Absolute structure: Flack *x* determined using 2632 quotients [(I+)-(I-)]/[(I+)+(I-)] (Parsons *et al.*, 2013)
1 restraint	Absolute structure parameter: 0.05 (8)

### Special details

**Table:**

Geometry. All e.s.d.'s (except the e.s.d. in the dihedral angle between two l.s. planes) are estimated using the full covariance matrix. The cell e.s.d.'s are taken into account individually in the estimation of e.s.d.'s in distances, angles and torsion angles; correlations between e.s.d.'s in cell parameters are only used when they are defined by crystal symmetry. An approximate (isotropic) treatment of cell e.s.d.'s is used for estimating e.s.d.'s involving l.s. planes.
Refinement. Refined as a 2-component twin.

### Fractional atomic coordinates and isotropic or equivalent isotropic displacement parameters (Å^2^)

**Table:**

	*x*	*y*	*z*	*U*_iso_*/*U*_eq_	
C1	1.1431 (5)	0.8972 (6)	0.9253 (3)	0.0153 (10)	
N2	1.1842 (4)	0.7850 (5)	0.8817 (2)	0.0163 (9)	
C3	1.1862 (5)	0.8314 (6)	0.8053 (3)	0.0149 (10)	
C4	1.1266 (5)	0.9782 (6)	0.8025 (3)	0.0143 (10)	
C5	1.0975 (5)	1.0166 (5)	0.8717 (3)	0.0135 (9)	
C6	1.0311 (5)	1.1589 (6)	0.8845 (3)	0.0159 (10)	
H6	1.0984	1.2269	0.9115	0.019*	
C7	0.9703 (5)	1.2250 (6)	0.8039 (3)	0.0167 (10)	
C8	1.0752 (5)	1.2122 (5)	0.7508 (3)	0.0144 (10)	
H8	1.1580	1.2596	0.7765	0.017*	
C9	1.0343 (5)	1.2809 (6)	0.6718 (3)	0.0194 (11)	
H9A	1.0006	1.3791	0.6779	0.023*	
H9B	0.9620	1.2237	0.6426	0.023*	
C10	1.1482 (5)	1.2899 (7)	0.6264 (3)	0.0231 (11)	
H10A	1.1855	1.1924	0.6231	0.028*	
H10B	1.2183	1.3519	0.6543	0.028*	
C11	1.1059 (7)	1.3504 (8)	0.5454 (4)	0.0349 (15)	
H11A	1.0380	1.2864	0.5171	0.042*	
H11B	1.0657	1.4463	0.5487	0.042*	
C12	1.2195 (8)	1.3646 (9)	0.5007 (4)	0.0433 (19)	
H12A	1.2627	1.2697	0.4998	0.052*	
H12B	1.2852	1.4326	0.5278	0.052*	
C13	1.1771 (12)	1.4175 (13)	0.4187 (5)	0.080 (4)	
H13A	1.1119	1.3511	0.3913	0.120*	
H13B	1.2538	1.4211	0.3936	0.120*	
H13C	1.1387	1.5138	0.4189	0.120*	
C14	1.2311 (5)	0.7567 (6)	0.7508 (3)	0.0213 (11)	
H14A	1.2660	0.6629	0.7611	0.026*	
H14B	1.2277	0.7979	0.7021	0.026*	
C15	0.8381 (5)	1.1508 (6)	0.7705 (3)	0.0193 (10)	
H15A	0.8540	1.0495	0.7600	0.029*	
H15B	0.7986	1.1992	0.7235	0.029*	
H15C	0.7782	1.1570	0.8070	0.029*	
C16	1.2068 (6)	0.5407 (6)	0.9208 (3)	0.0203 (11)	
H16A	1.1648	0.5103	0.8701	0.031*	
H16B	1.1400	0.5521	0.9528	0.031*	
H16C	1.2706	0.4678	0.9429	0.031*	
O1	1.1050 (4)	1.0568 (4)	0.73717 (19)	0.0172 (7)	
O2	0.9541 (4)	1.3789 (4)	0.8112 (2)	0.0183 (8)	
H2	0.8942	1.3951	0.8359	0.027*	
O3	0.9324 (4)	1.1327 (4)	0.9312 (2)	0.0213 (8)	
H3	0.9060	1.2114	0.9456	0.032*	
O4	1.1447 (4)	0.8881 (4)	0.99543 (19)	0.0183 (8)	
O5	1.2747 (4)	0.6794 (4)	0.9159 (2)	0.0194 (8)	
C1A	0.4815 (5)	1.9716 (6)	0.8996 (3)	0.0156 (10)	
N2A	0.4361 (4)	2.0638 (5)	0.8379 (2)	0.0174 (9)	
C3A	0.4890 (5)	2.0271 (6)	0.7717 (3)	0.0159 (10)	
C4A	0.5564 (5)	1.8865 (6)	0.7938 (3)	0.0136 (9)	
C5A	0.5529 (5)	1.8520 (5)	0.8678 (3)	0.0147 (10)	
C6A	0.6116 (5)	1.7116 (5)	0.9032 (3)	0.0140 (10)	
H6A	0.5393	1.6398	0.9020	0.017*	
C7A	0.7097 (5)	1.6545 (5)	0.8525 (3)	0.0126 (9)	
C8A	0.6355 (5)	1.6563 (5)	0.7676 (3)	0.0140 (10)	
H8A	0.5498	1.6055	0.7656	0.017*	
C9A	0.7086 (5)	1.5840 (6)	0.7096 (3)	0.0191 (11)	
H9AA	0.7419	1.4891	0.7296	0.023*	
H9AB	0.7853	1.6439	0.7038	0.023*	
C10A	0.6212 (6)	1.5621 (7)	0.6302 (3)	0.0223 (11)	
H10C	0.5384	1.5139	0.6364	0.027*	
H10D	0.5986	1.6572	0.6066	0.027*	
C11A	0.6921 (6)	1.4697 (7)	0.5771 (3)	0.0270 (13)	
H11C	0.7732	1.5202	0.5699	0.032*	
H11D	0.7183	1.3768	0.6023	0.032*	
C12A	0.6074 (6)	1.4392 (8)	0.4983 (3)	0.0295 (14)	
H12C	0.5805	1.5318	0.4730	0.035*	
H12D	0.5269	1.3871	0.5051	0.035*	
C13A	0.6823 (7)	1.3483 (8)	0.4466 (3)	0.0344 (15)	
H13D	0.6244	1.3297	0.3982	0.052*	
H13E	0.7096	1.2566	0.4715	0.052*	
H13F	0.7599	1.4013	0.4377	0.052*	
C14A	0.4806 (5)	2.1038 (6)	0.7075 (3)	0.0210 (11)	
H14C	0.4356	2.1932	0.7026	0.025*	
H14D	0.5198	2.0685	0.6671	0.025*	
C15A	0.8399 (5)	1.7422 (6)	0.8646 (3)	0.0160 (10)	
H15D	0.8201	1.8444	0.8550	0.024*	
H15E	0.8958	1.7077	0.8296	0.024*	
H15F	0.8858	1.7298	0.9168	0.024*	
C16A	0.5035 (6)	2.3002 (6)	0.8752 (4)	0.0273 (12)	
H16D	0.5616	2.2998	0.8376	0.041*	
H16E	0.5520	2.2663	0.9238	0.041*	
H16F	0.4719	2.3983	0.8809	0.041*	
O1A	0.6069 (4)	1.8064 (4)	0.74144 (18)	0.0166 (7)	
O2A	0.7369 (3)	1.5024 (4)	0.86892 (19)	0.0145 (7)	
H2A	0.7410	1.4877	0.9152	0.022*	
O3A	0.6775 (3)	1.7297 (4)	0.98091 (18)	0.0145 (7)	
H3A	0.6363	1.6850	1.0097	0.022*	
O4A	0.4586 (4)	1.9909 (4)	0.9650 (2)	0.0179 (7)	
O5A	0.3911 (4)	2.2045 (4)	0.8499 (2)	0.0186 (8)	

### Atomic displacement parameters (Å^2^)

**Table:**

	*U*^11^	*U*^22^	*U*^33^	*U*^12^	*U*^13^	*U*^23^
C1	0.015 (2)	0.013 (2)	0.017 (2)	−0.004 (2)	−0.0009 (19)	−0.0023 (19)
N2	0.016 (2)	0.013 (2)	0.018 (2)	0.0036 (17)	−0.0009 (16)	0.0025 (17)
C3	0.011 (2)	0.018 (2)	0.016 (2)	−0.0028 (19)	0.0039 (18)	0.0019 (19)
C4	0.014 (2)	0.016 (2)	0.015 (2)	−0.0008 (19)	0.0064 (18)	−0.0002 (19)
C5	0.010 (2)	0.014 (2)	0.017 (2)	−0.0018 (19)	0.0024 (17)	−0.0027 (19)
C6	0.017 (2)	0.017 (2)	0.014 (2)	0.001 (2)	0.0063 (19)	−0.0035 (19)
C7	0.017 (2)	0.012 (2)	0.022 (3)	0.001 (2)	0.006 (2)	−0.0002 (19)
C8	0.013 (2)	0.012 (2)	0.017 (2)	0.0003 (18)	−0.0002 (18)	0.0007 (19)
C9	0.018 (2)	0.021 (3)	0.018 (2)	0.005 (2)	0.0005 (19)	0.006 (2)
C10	0.022 (3)	0.027 (3)	0.020 (3)	0.009 (2)	0.003 (2)	0.008 (2)
C11	0.037 (4)	0.041 (4)	0.027 (3)	0.016 (3)	0.009 (3)	0.017 (3)
C12	0.055 (5)	0.047 (4)	0.035 (3)	0.015 (4)	0.027 (3)	0.021 (3)
C13	0.113 (9)	0.095 (8)	0.046 (5)	0.054 (7)	0.049 (5)	0.046 (5)
C14	0.021 (3)	0.022 (3)	0.022 (3)	0.005 (2)	0.009 (2)	−0.002 (2)
C15	0.016 (2)	0.020 (3)	0.021 (2)	0.000 (2)	0.003 (2)	0.000 (2)
C16	0.024 (3)	0.012 (2)	0.026 (3)	0.000 (2)	0.007 (2)	0.000 (2)
O1	0.0200 (18)	0.0170 (18)	0.0144 (16)	0.0042 (15)	0.0022 (13)	−0.0008 (14)
O2	0.0174 (18)	0.0135 (17)	0.0253 (19)	0.0032 (14)	0.0075 (15)	0.0004 (14)
O3	0.030 (2)	0.0158 (18)	0.0222 (18)	0.0035 (16)	0.0158 (16)	−0.0017 (15)
O4	0.0252 (19)	0.0170 (17)	0.0123 (16)	−0.0038 (15)	0.0022 (14)	0.0014 (14)
O5	0.0177 (18)	0.0127 (18)	0.0263 (19)	0.0023 (14)	−0.0006 (15)	0.0034 (14)
C1A	0.013 (2)	0.013 (2)	0.021 (2)	−0.0010 (19)	0.0022 (19)	−0.0025 (19)
N2A	0.021 (2)	0.011 (2)	0.020 (2)	0.0053 (18)	0.0049 (17)	−0.0022 (17)
C3A	0.010 (2)	0.016 (2)	0.023 (2)	−0.0024 (19)	0.0038 (18)	−0.005 (2)
C4A	0.010 (2)	0.014 (2)	0.017 (2)	−0.0044 (19)	0.0032 (18)	−0.0027 (19)
C5A	0.013 (2)	0.014 (2)	0.019 (2)	−0.0059 (19)	0.0059 (19)	−0.0034 (19)
C6A	0.016 (2)	0.014 (2)	0.012 (2)	−0.0061 (19)	0.0038 (18)	−0.0016 (18)
C7A	0.012 (2)	0.011 (2)	0.015 (2)	0.0008 (19)	0.0008 (18)	0.0019 (18)
C8A	0.014 (2)	0.014 (2)	0.013 (2)	0.0007 (19)	−0.0007 (18)	0.0015 (18)
C9A	0.018 (3)	0.024 (3)	0.015 (2)	0.006 (2)	0.002 (2)	0.000 (2)
C10A	0.023 (3)	0.028 (3)	0.016 (2)	0.006 (2)	0.004 (2)	−0.005 (2)
C11A	0.031 (3)	0.032 (3)	0.018 (3)	0.010 (3)	0.003 (2)	−0.004 (2)
C12A	0.029 (3)	0.041 (4)	0.019 (3)	0.006 (3)	0.003 (2)	−0.008 (2)
C13A	0.040 (4)	0.043 (4)	0.020 (3)	0.003 (3)	0.005 (3)	−0.010 (3)
C14A	0.021 (3)	0.021 (3)	0.021 (3)	0.005 (2)	0.005 (2)	0.003 (2)
C15A	0.017 (2)	0.018 (2)	0.014 (2)	−0.002 (2)	0.0056 (18)	0.0015 (19)
C16A	0.029 (3)	0.017 (3)	0.036 (3)	−0.011 (2)	0.006 (2)	−0.008 (2)
O1A	0.0188 (17)	0.0195 (18)	0.0120 (15)	0.0060 (15)	0.0039 (13)	0.0011 (14)
O2A	0.0181 (17)	0.0128 (17)	0.0122 (15)	0.0024 (14)	0.0016 (13)	0.0016 (13)
O3A	0.0175 (17)	0.0154 (17)	0.0104 (15)	−0.0013 (14)	0.0022 (13)	0.0008 (13)
O4A	0.0234 (19)	0.0152 (18)	0.0175 (17)	0.0020 (15)	0.0105 (14)	−0.0016 (14)
O5A	0.0157 (18)	0.0102 (17)	0.0301 (19)	0.0022 (14)	0.0044 (15)	−0.0040 (15)

### Geometric parameters (Å, º)

**Table:**

C1—N2	1.385 (7)	C1A—N2A	1.386 (7)
C1—C5	1.460 (7)	C1A—C5A	1.472 (7)
C1—O4	1.234 (6)	C1A—O4A	1.226 (6)
N2—C3	1.412 (6)	N2A—C3A	1.406 (7)
N2—O5	1.391 (5)	N2A—O5A	1.389 (5)
C3—C4	1.463 (7)	C3A—C4A	1.471 (7)
C3—C14	1.320 (7)	C3A—C14A	1.318 (7)
C4—C5	1.349 (7)	C4A—C5A	1.347 (7)
C4—O1	1.339 (6)	C4A—O1A	1.345 (6)
C5—C6	1.495 (7)	C5A—C6A	1.498 (7)
C6—H6	0.9800	C6A—H6A	0.9800
C6—C7	1.563 (7)	C6A—C7A	1.540 (7)
C6—O3	1.423 (6)	C6A—O3A	1.422 (6)
C7—C8	1.540 (7)	C7A—C8A	1.549 (6)
C7—C15	1.523 (7)	C7A—C15A	1.526 (7)
C7—O2	1.419 (6)	C7A—O2A	1.431 (6)
C8—H8	0.9800	C8A—H8A	0.9800
C8—C9	1.514 (7)	C8A—C9A	1.515 (7)
C8—O1	1.475 (6)	C8A—O1A	1.455 (6)
C9—H9A	0.9700	C9A—H9AA	0.9700
C9—H9B	0.9700	C9A—H9AB	0.9700
C9—C10	1.518 (7)	C9A—C10A	1.529 (7)
C10—H10A	0.9700	C10A—H10C	0.9700
C10—H10B	0.9700	C10A—H10D	0.9700
C10—C11	1.519 (7)	C10A—C11A	1.528 (7)
C11—H11A	0.9700	C11A—H11C	0.9700
C11—H11B	0.9700	C11A—H11D	0.9700
C11—C12	1.510 (9)	C11A—C12A	1.523 (8)
C12—H12A	0.9700	C12A—H12C	0.9700
C12—H12B	0.9700	C12A—H12D	0.9700
C12—C13	1.511 (10)	C12A—C13A	1.527 (8)
C13—H13A	0.9600	C13A—H13D	0.9600
C13—H13B	0.9600	C13A—H13E	0.9600
C13—H13C	0.9600	C13A—H13F	0.9600
C14—H14A	0.9300	C14A—H14C	0.9300
C14—H14B	0.9300	C14A—H14D	0.9300
C15—H15A	0.9600	C15A—H15D	0.9600
C15—H15B	0.9600	C15A—H15E	0.9600
C15—H15C	0.9600	C15A—H15F	0.9600
C16—H16A	0.9600	C16A—H16D	0.9600
C16—H16B	0.9600	C16A—H16E	0.9600
C16—H16C	0.9600	C16A—H16F	0.9600
C16—O5	1.448 (6)	C16A—O5A	1.442 (6)
O2—H2	0.8200	O2A—H2A	0.8200
O3—H3	0.8200	O3A—H3A	0.8200
			
N2—C1—C5	106.4 (4)	N2A—C1A—C5A	105.5 (4)
O4—C1—N2	123.6 (5)	O4A—C1A—N2A	123.9 (5)
O4—C1—C5	129.9 (5)	O4A—C1A—C5A	130.6 (5)
C1—N2—C3	111.3 (4)	C1A—N2A—C3A	112.5 (4)
C1—N2—O5	120.6 (4)	C1A—N2A—O5A	120.8 (4)
O5—N2—C3	120.0 (4)	O5A—N2A—C3A	122.0 (4)
N2—C3—C4	103.3 (4)	N2A—C3A—C4A	102.4 (4)
C14—C3—N2	127.0 (5)	C14A—C3A—N2A	127.4 (5)
C14—C3—C4	129.7 (5)	C14A—C3A—C4A	130.1 (5)
C5—C4—C3	111.2 (4)	C5A—C4A—C3A	111.6 (4)
O1—C4—C3	121.4 (4)	O1A—C4A—C3A	120.4 (4)
O1—C4—C5	127.4 (5)	O1A—C4A—C5A	127.9 (5)
C1—C5—C6	130.4 (4)	C1A—C5A—C6A	131.2 (4)
C4—C5—C1	107.1 (4)	C4A—C5A—C1A	107.1 (4)
C4—C5—C6	122.5 (5)	C4A—C5A—C6A	121.6 (4)
C5—C6—H6	109.0	C5A—C6A—H6A	108.7
C5—C6—C7	108.3 (4)	C5A—C6A—C7A	107.3 (4)
C7—C6—H6	109.0	C7A—C6A—H6A	108.7
O3—C6—C5	108.9 (4)	O3A—C6A—C5A	112.7 (4)
O3—C6—H6	109.0	O3A—C6A—H6A	108.7
O3—C6—C7	112.6 (4)	O3A—C6A—C7A	110.8 (4)
C8—C7—C6	108.5 (4)	C6A—C7A—C8A	107.6 (4)
C15—C7—C6	111.0 (4)	C15A—C7A—C6A	112.0 (4)
C15—C7—C8	112.8 (4)	C15A—C7A—C8A	113.0 (4)
O2—C7—C6	109.4 (4)	O2A—C7A—C6A	109.3 (4)
O2—C7—C8	103.5 (4)	O2A—C7A—C8A	104.4 (4)
O2—C7—C15	111.4 (4)	O2A—C7A—C15A	110.3 (4)
C7—C8—H8	108.3	C7A—C8A—H8A	107.8
C9—C8—C7	114.8 (4)	C9A—C8A—C7A	115.7 (4)
C9—C8—H8	108.3	C9A—C8A—H8A	107.8
O1—C8—C7	110.8 (4)	O1A—C8A—C7A	110.5 (4)
O1—C8—H8	108.3	O1A—C8A—H8A	107.8
O1—C8—C9	106.1 (4)	O1A—C8A—C9A	106.8 (4)
C8—C9—H9A	108.9	C8A—C9A—H9AA	108.9
C8—C9—H9B	108.9	C8A—C9A—H9AB	108.9
C8—C9—C10	113.3 (4)	C8A—C9A—C10A	113.5 (4)
H9A—C9—H9B	107.7	H9AA—C9A—H9AB	107.7
C10—C9—H9A	108.9	C10A—C9A—H9AA	108.9
C10—C9—H9B	108.9	C10A—C9A—H9AB	108.9
C9—C10—H10A	108.9	C9A—C10A—H10C	109.3
C9—C10—H10B	108.9	C9A—C10A—H10D	109.3
C9—C10—C11	113.5 (4)	H10C—C10A—H10D	107.9
H10A—C10—H10B	107.7	C11A—C10A—C9A	111.7 (5)
C11—C10—H10A	108.9	C11A—C10A—H10C	109.3
C11—C10—H10B	108.9	C11A—C10A—H10D	109.3
C10—C11—H11A	108.8	C10A—C11A—H11C	108.7
C10—C11—H11B	108.8	C10A—C11A—H11D	108.7
H11A—C11—H11B	107.7	H11C—C11A—H11D	107.6
C12—C11—C10	113.9 (5)	C12A—C11A—C10A	114.0 (5)
C12—C11—H11A	108.8	C12A—C11A—H11C	108.7
C12—C11—H11B	108.8	C12A—C11A—H11D	108.7
C11—C12—H12A	108.7	C11A—C12A—H12C	109.1
C11—C12—H12B	108.7	C11A—C12A—H12D	109.1
C11—C12—C13	114.2 (7)	C11A—C12A—C13A	112.4 (5)
H12A—C12—H12B	107.6	H12C—C12A—H12D	107.8
C13—C12—H12A	108.7	C13A—C12A—H12C	109.1
C13—C12—H12B	108.7	C13A—C12A—H12D	109.1
C12—C13—H13A	109.5	C12A—C13A—H13D	109.5
C12—C13—H13B	109.5	C12A—C13A—H13E	109.5
C12—C13—H13C	109.5	C12A—C13A—H13F	109.5
H13A—C13—H13B	109.5	H13D—C13A—H13E	109.5
H13A—C13—H13C	109.5	H13D—C13A—H13F	109.5
H13B—C13—H13C	109.5	H13E—C13A—H13F	109.5
C3—C14—H14A	120.0	C3A—C14A—H14C	120.0
C3—C14—H14B	120.0	C3A—C14A—H14D	120.0
H14A—C14—H14B	120.0	H14C—C14A—H14D	120.0
C7—C15—H15A	109.5	C7A—C15A—H15D	109.5
C7—C15—H15B	109.5	C7A—C15A—H15E	109.5
C7—C15—H15C	109.5	C7A—C15A—H15F	109.5
H15A—C15—H15B	109.5	H15D—C15A—H15E	109.5
H15A—C15—H15C	109.5	H15D—C15A—H15F	109.5
H15B—C15—H15C	109.5	H15E—C15A—H15F	109.5
H16A—C16—H16B	109.5	H16D—C16A—H16E	109.5
H16A—C16—H16C	109.5	H16D—C16A—H16F	109.5
H16B—C16—H16C	109.5	H16E—C16A—H16F	109.5
O5—C16—H16A	109.5	O5A—C16A—H16D	109.5
O5—C16—H16B	109.5	O5A—C16A—H16E	109.5
O5—C16—H16C	109.5	O5A—C16A—H16F	109.5
C4—O1—C8	112.3 (4)	C4A—O1A—C8A	111.8 (4)
C7—O2—H2	109.5	C7A—O2A—H2A	109.5
C6—O3—H3	109.5	C6A—O3A—H3A	109.5
N2—O5—C16	110.1 (4)	N2A—O5A—C16A	109.9 (4)
			
C1—N2—C3—C4	−6.7 (5)	C1A—N2A—C3A—C4A	8.7 (5)
C1—N2—C3—C14	173.8 (5)	C1A—N2A—C3A—C14A	−170.0 (5)
C1—N2—O5—C16	107.4 (5)	C1A—N2A—O5A—C16A	76.8 (6)
C1—C5—C6—C7	−165.0 (5)	C1A—C5A—C6A—C7A	−162.9 (5)
C1—C5—C6—O3	−42.4 (7)	C1A—C5A—C6A—O3A	−40.7 (7)
N2—C1—C5—C4	−6.6 (5)	N2A—C1A—C5A—C4A	5.5 (5)
N2—C1—C5—C6	173.7 (5)	N2A—C1A—C5A—C6A	−172.8 (5)
N2—C3—C4—C5	2.4 (5)	N2A—C3A—C4A—C5A	−5.0 (5)
N2—C3—C4—O1	−176.8 (4)	N2A—C3A—C4A—O1A	172.2 (4)
C3—N2—O5—C16	−106.7 (5)	C3A—N2A—O5A—C16A	−77.1 (6)
C3—C4—C5—C1	2.5 (6)	C3A—C4A—C5A—C1A	−0.2 (6)
C3—C4—C5—C6	−177.7 (4)	C3A—C4A—C5A—C6A	178.2 (4)
C3—C4—O1—C8	−166.3 (4)	C3A—C4A—O1A—C8A	−164.3 (4)
C4—C5—C6—C7	15.3 (7)	C4A—C5A—C6A—C7A	19.1 (6)
C4—C5—C6—O3	137.9 (5)	C4A—C5A—C6A—O3A	141.3 (4)
C5—C1—N2—C3	8.4 (6)	C5A—C1A—N2A—C3A	−9.1 (6)
C5—C1—N2—O5	157.1 (4)	C5A—C1A—N2A—O5A	−165.3 (4)
C5—C4—O1—C8	14.7 (7)	C5A—C4A—O1A—C8A	12.4 (7)
C5—C6—C7—C8	−45.3 (5)	C5A—C6A—C7A—C8A	−49.7 (5)
C5—C6—C7—C15	79.3 (5)	C5A—C6A—C7A—C15A	75.0 (5)
C5—C6—C7—O2	−157.4 (4)	C5A—C6A—C7A—O2A	−162.5 (4)
C6—C7—C8—C9	−176.2 (4)	C6A—C7A—C8A—C9A	−171.8 (4)
C6—C7—C8—O1	63.7 (5)	C6A—C7A—C8A—O1A	66.7 (5)
C7—C8—C9—C10	170.0 (5)	C7A—C8A—C9A—C10A	168.8 (4)
C7—C8—O1—C4	−47.0 (5)	C7A—C8A—O1A—C4A	−45.8 (5)
C8—C9—C10—C11	176.6 (5)	C8A—C9A—C10A—C11A	−171.9 (5)
C9—C8—O1—C4	−172.2 (4)	C9A—C8A—O1A—C4A	−172.5 (4)
C9—C10—C11—C12	178.0 (6)	C9A—C10A—C11A—C12A	177.7 (5)
C10—C11—C12—C13	177.0 (8)	C10A—C11A—C12A—C13A	179.3 (6)
C14—C3—C4—C5	−178.1 (5)	C14A—C3A—C4A—C5A	173.7 (5)
C14—C3—C4—O1	2.7 (8)	C14A—C3A—C4A—O1A	−9.1 (8)
C15—C7—C8—C9	60.4 (6)	C15A—C7A—C8A—C9A	64.2 (6)
C15—C7—C8—O1	−59.7 (5)	C15A—C7A—C8A—O1A	−57.4 (5)
O1—C4—C5—C1	−178.4 (5)	O1A—C4A—C5A—C1A	−177.1 (5)
O1—C4—C5—C6	1.4 (8)	O1A—C4A—C5A—C6A	1.3 (8)
O1—C8—C9—C10	−67.3 (6)	O1A—C8A—C9A—C10A	−67.7 (6)
O2—C7—C8—C9	−60.1 (5)	O2A—C7A—C8A—C9A	−55.7 (5)
O2—C7—C8—O1	179.8 (4)	O2A—C7A—C8A—O1A	−177.2 (4)
O3—C6—C7—C8	−165.6 (4)	O3A—C6A—C7A—C8A	−173.1 (4)
O3—C6—C7—C15	−41.1 (6)	O3A—C6A—C7A—C15A	−48.4 (5)
O3—C6—C7—O2	82.2 (5)	O3A—C6A—C7A—O2A	74.1 (5)
O4—C1—N2—C3	−173.2 (5)	O4A—C1A—N2A—C3A	173.0 (5)
O4—C1—N2—O5	−24.5 (7)	O4A—C1A—N2A—O5A	16.8 (8)
O4—C1—C5—C4	175.2 (5)	O4A—C1A—C5A—C4A	−176.8 (5)
O4—C1—C5—C6	−4.6 (9)	O4A—C1A—C5A—C6A	5.0 (9)
O5—N2—C3—C4	−155.6 (4)	O5A—N2A—C3A—C4A	164.6 (4)
O5—N2—C3—C14	24.9 (8)	O5A—N2A—C3A—C14A	−14.2 (8)

### Hydrogen-bond geometry (Å, º)

**Table:**

*D*—H···*A*	*D*—H	H···*A*	*D*···*A*	*D*—H···*A*
O2—H2···O2*A*	0.82	2.04	2.818 (5)	158
O3—H3···O4^i^	0.82	2.03	2.836 (5)	168
O2*A*—H2*A*···O4^i^	0.82	2.00	2.685 (5)	141
O3*A*—H3*A*···O4*A*^ii^	0.82	2.10	2.829 (5)	149

Symmetry codes: (i) −*x*+2, *y*+1/2, −*z*+2; (ii) −*x*+1, *y*−1/2, −*z*+2.
