# Supplementary figures and images for: Crystal structure of natural phaeosphaeride A
Source: Acta Crystallogr E Crystallogr Commun. 2015 Jul 31;71(Pt 8):o625–6. doi: 10.1107/S205698901501395X (PMC4571431; doi:10.1107/S205698901501395X)

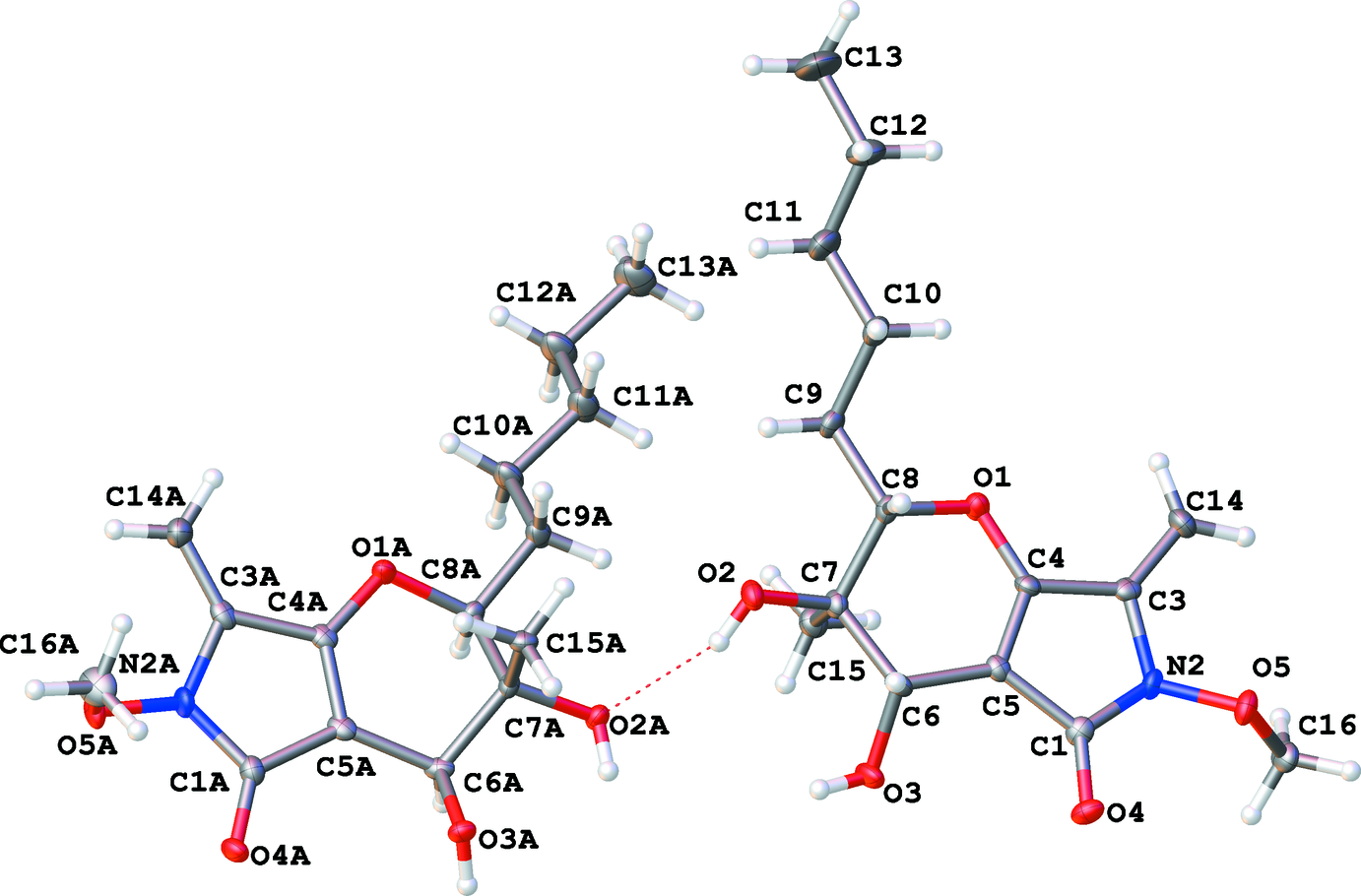

Supplement: Supplementary file 4 [file e-71-0o625-fig1.tif]

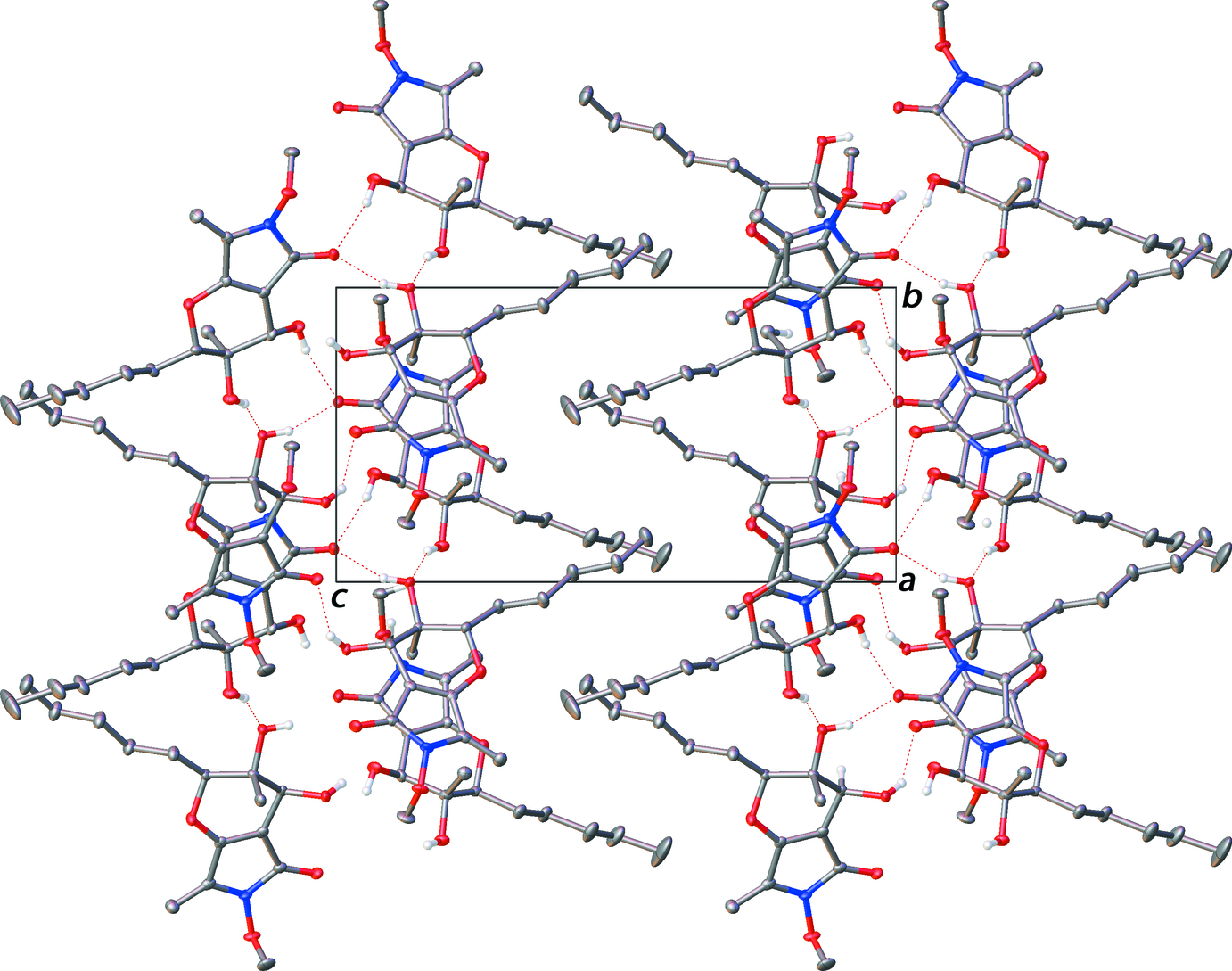

Supplement: Supplementary file 5 [file e-71-0o625-fig2.tif]
